# Supplementary material for: Exploring the clinical profiles and management of juvenile dermatomyositis in Africa: a survey of African rheumatology care providers
Source: Pediatr Rheumatol Online J. 2025 Jan 27;23:10. doi: 10.1186/s12969-024-01009-8 (PMC11771122; doi:10.1186/s12969-024-01009-8)
Supplement: Supplementary file 1 — Supplementary Material 1 [file 12969_2024_1009_MOESM1_ESM.docx]

Supplementary File 1: Juvenile dermatomyositis in Africa Survey

Start of Block: Introduction and background

We kindly request your participation in this survey to better understand the disease burden of juvenile dermatomyositis (JDM) in Africa. Currently, there are few published reports of JDM in African countries, which we suspect is related to underreporting rather than absence of disease. These reports suggest severe disease phenotypes, however the full spectrum of disease is currently unknown. We aim to use the results of this survey to better understand disease burden of JDM in African countries and compare it to published reports on JDM in Africa. We hope this will highlight the need to increase resources for diagnosis and treatment of patients with JDM in Africa.

We are also planning to conduct a comparative study between JDM patients in South Africa and Kenya and a large established cohort of JDM patients in North America. We hope to ultimately expand this work to include more countries throughout Africa in the future. Our primary aims are to 1) understand the current landscape of JDM in Africa, 2) identify disparities in JDM severity and outcomes, 3) understand barriers to diagnosis and care of children with JDM in Africa, and 4) improve recognition of JDM and access to diagnosis and treatment of JDM in Africa.

We hope you will be interested in collaborating with us on this project.

We estimate that this survey will take you 10-15 minutes to complete. You can stop at any time and return to the survey at a later time.

Please fill out this survey only once, even if you receive the survey link multiple times.

Since we hope to publish at least some of the findings from this survey, we are asking for your consent to participate in this survey. No names or other identifying information will be included in any publications or presentations, and your responses to this survey will remain confidential.

- I consent to participate in this survey (1)
- I do not consent to participate in this survey (2)

Q1 Do you see patients with JDM?

- Yes (1)
- No (2)

Q2 Where do you practice (please include city and country)? Please write "I prefer not to answer" if you do not want to share this information.

________________________________________________________________

Q3 What setting do you practice in? Please select all that apply.

- Academic hospital (1)
- Community hospital (2)
- Private clinic (3)
- Other (please describe) (4) __________________________________________________

Q4 What is the name of the institution at which you practice? Please skip if you prefer to not answer.

________________________________________________________________

Q5 Please indicate your primary role.

- Attending/staff adult rheumatologist (1)
- Attending/staff paediatric rheumatologist (2)
- Fellow in adult rheumatology (3)
- Fellow in paediatric rheumatology (4)
- General practitioner (5)
- General paediatrician (6)
- Other (please describe) (7) __________________________________________________

Q6 For the following questions, we wish to identify the TOTAL number of JDM patients followed at your center. Please consider if you and the other providers at your center SHARE or individually follow JDM patients. 

If you and other providers at your center SHARE patients, then our preference is for only one provider from your center to fill out this survey. If this is not easy to arrange, then please go ahead and individually complete the survey.

Please indicate how you will complete this survey:

- **I am answering this survey individually**; I am the only provider at my center who sees JDM patients. I individually follow my patients and do NOT share them with any other providers. (1)
- **I am answering this survey individually**; I am NOT the only provider at my center who sees JDM patients, and other providers at my center may also be individually completing this survey. For the rest of the survey, all my responses will represent just patients that I follow but may not be reflective of all patients at my center. (2)
- **I coordinated with providers at my center;** I am NOT the only provider at my center who sees JDM patients, but I am the only person completing this survey for our center. For the rest of the survey, all my responses will represent the patients for our whole center. (3)

End of Block: Introduction and background

Start of Block: Current JDM

Q7 The following section will ask you about patients with JDM who are CURRENTLY being followed by you/your center. They will be referred to as CURRENT JDM patients.

 If you are unsure of exact numbers, please provide your best estimate. If you are unsure how to answer the question, please write "I'm not sure".

Q8 How many patients with JDM are CURRENTLY being followed by you/your center? 

If you are unsure of exact numbers for any of these questions, please provide your best estimate. If you are unsure how to answer the question, please write "I'm not sure".

________________________________________________________________

Q9 How many CURRENT JDM patients have clinically inactive disease ON medication? 

*For the remainder of this survey, “clinically inactive disease” will be defined as: lack of evidence of myositis disease activity as assessed by global and extramuscular assessments, stable muscle strength and function, and normal muscle enzyme levels, per the International Myositis Assessment & Clinical Studies (IMACS) criteria for lack of evidence of active myositis.*

________________________________________________________________

Q10 How many CURRENT JDM patients are in remission OFF medication?

*For the remainder of the survey, “remission” will be defined according to the IMACS 2005 definition: clinically inactive disease while not receiving any drug therapy for a 6-month continuous period.*

________________________________________________________________

Q11 How many of the CURRENT JDM patients have developed calcinosis at some point during their disease?

________________________________________________________________

Q12 How many of the CURRENT JDM patients have developed interstitial lung disease (ILD) at some point during their disease?

________________________________________________________________

End of Block: Current JDM

Start of Block: Prior JDM

Q13 The following section will ask you about patients with JDM who had been followed by you/your center **in the past 10 years but are no longer being followed**. They will be referred to as PRIOR JDM patients.

 If you are unsure of exact numbers, please provide your best estimate. If you are unsure how to answer the question, please write "I'm not sure".

Q14 How many patients with JDM had been followed by you/your center in the past 10 years, but are no longer being followed by you/your center?

If you are unsure of exact numbers for any of these questions, please provide your best estimate. If you are unsure how to answer the question, please write "I'm not sure".

________________________________________________________________

Q15 How many PRIOR JDM patients achieved clinically inactive disease ON medication?

*As a reminder, “clinically inactive disease” is defined as: lack of evidence of myositis disease activity as assessed by global and extramuscular assessments, stable muscle strength and function, and normal muscle enzyme levels, per the International Myositis Assessment & Clinical Studies (IMACS) criteria for lack of evidence of active myositis.*

________________________________________________________________

Q16 How many PRIOR JDM patients achieved remission OFF medication?

*As a reminder, “remission” is defined according to the IMACS 2005 definition: clinically inactive disease while not receiving any drug therapy for a 6-month continuous period.*

________________________________________________________________

Q17 How many of the PRIOR JDM patients developed calcinosis at some point during their disease?

________________________________________________________________

Q18 How many of the PRIOR JDM patients developed interstitial lung disease (ILD) at some point during their disease?

________________________________________________________________

Q19 How many of the PRIOR JDM patients died?

________________________________________________________________

Q20 What are reasons these PRIOR JDM patients are no longer being followed by you/your center? Please select all that apply.

- Moved (1)
- Aged out and/or referred to adult provider (2)
- Lost to follow-up (3)
- Could no longer afford medical care (4)
- Passed away (5)
- Other (please describe) (6) __________________________________________________

End of Block: Prior JDM

Start of Block: Diagnosis and Treatment

Q21 Putting aside access and/or cost-related issues, which tools are AVAILABLE to diagnose and/or monitor JDM at your center? Please select all that apply.

- Inflammatory markers, including erythrocyte sedimentation rate (ESR) and/or c-reactive protein (CRP) (1)
- Muscle enzymes, including creatine kinase (CK), aldolase, lactate dehydrogenase (LDH), and/or aspartate aminotransferase (AST) (2)
- von Willebrand factor antigen (3)
- Antinuclear antibodies (4)
- Myositis-specific or myositis-associated antibodies, including ANY of the following: **Myositis-specific antibodies** include: Mi-2, MDA5 (CADM140), NXP-2 (MJ), TIF1 (p155/140), SRP, anti-synthetase (Jo-1, PL-7, PL-12, EJ, OJ, KS, Zo, Ha, YRS) **Myositis-associated antibodies** include: Pm-Scl, U1RNP, U1/U2RNP, U3RNP, Ro, La, Ku  (5)
- Muscle biopsy (6)
- Electromyography (EMG) (7)
- Disease assessment tools, including ANY of the following: Childhood Myositis Assessment Scale (CMAS), Childhood Health Assessment Questionnaire (CHAQ), Manual Muscle Testing (MMT), Physician Global Activity Visual Analogue Scale (VAS), Patient/Parent Global Activity VAS, Myositis Disease Activity Assessment Tool (MDAAT), Myositis Disease Damage Index (MDI), Physician Global Assessment of Disease Damage, Patient/Parent Global Assessment of Disease Damage (8)
- Other (please specify) (9) __________________________________________________

Q22 Now considering access and/or cost-related issues, for the tools you indicated as having available in the previous question, which do you typically use to diagnose and/or monitor JDM? Please select all that apply.

- None of the above - please explain why not (1) __________________________________________________
- Inflammatory markers, including erythrocyte sedimentation rate (ESR) and/or c-reactive protein (CRP) (2)
- Muscle enzymes, including creatine kinase (CK), aldolase, lactate dehydrogenase (LDH), and/or aspartate aminotransferase (AST) (3)
- von Willebrand factor antigen (4)
- Antinuclear antibodies (5)
- Myositis-specific or myositis-associated antibodies, including ANY of the following: **Myositis-specific antibodies** include: Mi-2, MDA5 (CADM140), NXP-2 (MJ), TIF1 (p155/140), SRP, anti-synthetase (Jo-1, PL-7, PL-12, EJ, OJ, KS, Zo, Ha, YRS) **Myositis-associated antibodies** include: Pm-Scl, U1RNP, U1/U2RNP, U3RNP, Ro, La, Ku  (6)
- Muscle biopsy (7)
- Electromyography (EMG) (8)
- Disease assessment tools, including ANY of the following: Childhood Myositis Assessment Scale (CMAS), Childhood Health Assessment Questionnaire (CHAQ), Manual Muscle Testing (MMT), Physician Global Activity Visual Analogue Scale (VAS), Patient/Parent Global Activity VAS, Myositis Disease Activity Assessment Tool (MDAAT), Myositis Disease Damage Index (MDI), Physician Global Assessment of Disease Damage, Patient/Parent Global Assessment of Disease Damage (9)
- Other (please specify) (10)

Q23 Putting aside access and/or cost-related issues, which tools are AVAILABLE to diagnose and/or monitor calcinosis in JDM? Please select all that apply.

- Physical examination (1)
- X-rays (2)
- MRI (3)
- Ultrasound (4)
- Other (please specify) (5) __________________________________________________

Q24 Now considering access and/or cost-related issues, for the tools you indicated as having available in the previous question, which do you typically use to diagnose and/or monitor calcinosis in JDM? Please select all that apply.

- None of the above - please explain why not (1) __________________________________________________
- Physical examination (2)
- X-rays (3)
- MRI (4)
- Ultrasound (5)
- Other (please specify) (6)

Q25 Putting aside access and/or cost-related issues, which tools are AVAILABLE to diagnose and/or monitor interstitial lung disease (ILD) in JDM? Please select all that apply.

- X-rays (1)
- Computerized tomography (CT) scan (2)
- Pulmonary function test (PFT) (3)
- Other (please specify) (4) __________________________________________________

Q26 Now considering access and/or cost-related issues, for the tools you indicated as having available in the previous question, which do you typically use to diagnose and/or monitor interstitial lung disease (ILD) in JDM? Please select all that apply.

- None of the above - please explain why not (1) __________________________________________________
- X-rays (2)
- Computerized tomography (CT) scan (3)
- Pulmonary function test (PFT) (4)
- Other (please specify) (5)

Q27 Putting aside access and/or cost-related issues, which therapies are AVAILABLE to treat patients with JDM? Please select all that apply.

- Prednisone or prednisolone (1)
- Intravenous methylprednisolone pulse dosing (15-30mg/kg) (2)
- Hydroxychloroquine (3)
- Methotrexate (4)
- Intravenous immune globulin (IVIG) (5)
- Mycophenolate mofetil or Mycophenolic acid (6)
- Azathioprine (7)
- Tacrolimus (8)
- Cyclosporine or ciclosporin (9)
- Rituximab (10)
- Cyclophosphamide (11)
- Tumor necrosis factor (TNF) inhibitors (i.e. adalimumab, infliximab) (12)
- Abatacept (13)
- Janus kinase inhibitors (14)
- Other (please specify) (15) __________________________________________________

Q28 Now considering access and/or cost-related issues, for the therapies you indicated as having available in the previous question, which do you typically use to treat patients with JDM? Please select all that apply.

- None of the above - please explain why not (1) __________________________________________________
- Prednisone or prednisolone (2)
- Intravenous methylprednisolone pulse dosing (15-30mg/kg) (3)
- Hydroxychloroquine (4)
- Methotrexate (5)
- Intravenous immune globulin (IVIG) (6)
- Mycophenolate mofetil or Mycophenolic acid (7)
- Azathioprine (8)
- Tacrolimus (9)
- Cyclosporine or ciclosporin (10)
- Rituximab (11)
- Cyclophosphamide (12)
- Tumor necrosis factor (TNF) inhibitors (i.e. adalimumab, infliximab) (13)
- Abatacept (14)
- Janus kinase inhibitors (15)
- Other (please specify) (16)

Q29 What are challenges you face in managing children with JDM? Please select all that apply.

- Limited availability of diagnostic tools (1)
- Limited availability of medications (2)
- Limited availability of non-medical therapies (such as physical and occupational therapy) (3)
- Caregivers unfamiliar with JDM (4)
- Other medical providers unfamiliar with JDM (5)
- I have limited familiarity with JDM (6)
- Delayed presentation to care (7)
- Caregivers/patients unwilling to initiate/continue treatment due to stigma surrounding disease (8)
- Other (please describe) (9) __________________________________________________

End of Block: Diagnosis and Treatment

Start of Block: Conclusion

Q30 We are also interested in improving and standardizing data collection for children with JDM in Africa to allow for continued research efforts to ultimately improve health outcomes. Do you and/or your center use a Minimum Data Set (MDS) for children with JDM and/or other paediatric rheumatic diseases?

- Yes - for patients with JDM (1)
- Yes - for patients with paediatric rheumatic diseases other than JDM (please specify which diseases) (2) __________________________________________________
- No - I/we do not use an MDS for any paediatric rheumatic diseases (3)
- Other (4) __________________________________________________

Q31 Would you be willing to share your Minimum Data Set (MDS)? Your answer is non-binding.

- Yes (1)
- No (2)

Q32 Thank you for your willingness to collaborate and share your MDS. Please provide your name, email, and phone number if you agree to allow us to contact you regarding sharing your MDS. 

If you choose to participate in additional aspects of this project and provide us with your contact information, then your survey responses may no longer be anonymous to the researchers. However, as stated at the start of the survey, no names or other identifying information will be included in any publications or presentations, and your responses to this survey will remain confidential. If you have questions, or would like to discuss further, please email Jessica Perfetto at jperfetto@montefiore.org.

- Name (1) __________________________________________________
- Email (2) __________________________________________________
- Phone number (with country code) (3) __________________________________________________
- Please share any questions or comments: (4) __________________________________________________

Q33 Thank you for taking the time to answer this survey. Would you be interested in collaborating on future projects by collecting clinical data on children with JDM at your center? Your answer is non-binding.

- Yes (1)
- No (2)

Q34 Please provide your name, email, and/or phone number if you agree to allow us to contact you. If you choose to participate in additional aspects of this project and provide us with your contact information, then your survey responses may no longer be anonymous to the researchers. However, as stated at the start of the survey, no names or other identifying information will be included in any publications or presentations, and your responses to this survey will remain confidential.

If you have questions, or would like to discuss further, please email Jessica Perfetto at jperfetto@montefiore.org.

- Name (1) __________________________________________________
- Email (2) __________________________________________________
- Phone number (with country code) (3) __________________________________________________
- Please share any questions or comments: (4) __________________________________________________

End of Block: Conclusion
