# Supplementary material for: Exploring the clinical profiles and management of juvenile dermatomyositis in Africa: a survey of African rheumatology care providers
Source: Pediatr Rheumatol Online J. 2025 Jan 27;23:10. doi: 10.1186/s12969-024-01009-8 (PMC11771122; doi:10.1186/s12969-024-01009-8)
Supplement: Supplementary file 3 — Supplementary Material 3 [file 12969_2024_1009_MOESM3_ESM.docx]

**Exploring the clinical profiles and management of juvenile dermatomyositis in Africa: a survey of African rheumatology care providers**

**Authors:** Jessica Perfetto, MD^1^, Laura B. Lewandowski, MD, MS^2^, Dawn M. Wahezi, MD, MS^1^, Vanessa Ogega, MBChB^3^, Joan Ahimbisibwe, MD^3^, Kate Webb, MBBS, PhD^4^, Christiaan Scott, MBChB^4^, Angela Migowa, MBChB^3^

**Affiliations:**^1^The Children’s Hospital at Montefiore, Bronx, NY, USA; ^2^ Lupus Genomics and Global Health Disparities Unit, Systemic Autoimmunity Branch, National Institute of Arthritis and Musculoskeletal and Skin Diseases, NIH, Bethesda, MD, USA; ^3^Aga Khan University Medical College East Africa, Nairobi, Kenya; ^4^Red Cross War Memorial Children’s Hospital, Cape Town, South Africa

**Corresponding author: Jessica Perfetto**, The Children’s Hospital at Montefiore, Division of Rheumatology, 3334 Bainbridge Avenue, Bronx, NY, 10467. [jperfetto@montefiore.org](mailto:jperfetto@montefiore.org)

**Abstract**

**Background**

There are limited studies of juvenile dermatomyositis (JDM) in low and middle-income countries (LMIC). Many demonstrate delays to care, high prevalence of severe manifestations, and high mortality. Given the disease-associated damage with JDM, understanding JDM in Africa further is critical. Our objectives are to understand the burden of JDM in Africa and provider access to diagnostic tools and therapy through survey methodology.

**Methods**

A survey (available in English and French) was distributed via WhatsApp to 363 total members of the African League of Associations for Rheumatology (AFLAR; n=233) and Paediatric Society of the African League Against Rheumatism (PAFLAR; n=130) from November 2022-January 2023. Topics included respondent specialty, number of JDM patients followed, severe manifestations, and available diagnostic tools and medications (with and without considering cost).

**Results**

Forty-three (12%) of the 363 providers who received the survey started it. Among the 43 who started the survey, 37 (86%) provided consent and manage JDM patients; of these 37 providers, 4 (11%) partially and 16 (43%) fully completed the survey. Most were adult and/or pediatric rheumatologists (n=19; 95%). Respondents represented all 5 African regions and described 216 children with JDM within the last 10 years. There was high prevalence of calcinosis (as high as 100%) and interstitial lung disease (ILD) (as high as 32%); mortality rates in Kenya (6/42; 14%) and Zambia (2/7; 29%) exceeded the 1-3% mortality reported in studies of high-income countries. Thirteen of 27 diagnostic tools and medications were accessible to ≤50% of respondents after considering cost, mostly in Northern or Southern Africa (9/13; 69%). Despite being cost-free, disease assessment tools and physical exam to assess calcinosis were not reported as universally available or accessible.

**Conclusions**

This is the first study to explore experiences of providers caring for children with JDM in Africa. Respondents identified 216 children with JDM seen within the last 10 years, exceeding the 196 children with JDM reported within the last 25 years but likely still underestimating prevalence. Our findings align with reports of severe manifestations and poor outcomes in African children with JDM. Access to many diagnostics and medications is limited, and differences in accessibility parallel regional healthcare disparities. The potential differences in JDM severity warrant systematic study and highlight the need to include patients and providers from LMIC in collaborative research efforts.

**Keywords**: juvenile dermatomyositis, calcinosis, interstitial lung disease, low and middle-income countries, Africa

**Background**

There is a lack of data on pediatric rheumatic disease (PRD) in low and middle-income countries (LMIC) (1, 2, 3), and current reports are mostly limited to case studies or single center reports, with few national registries and no population-based studies (1). While this may falsely create a perception of rarity of PRD in LMIC, the paucity of data is likely attributable to limited diagnostic capacities, scarcity of pediatric rheumatologists (4, 5), and diagnostic confusion due to limited awareness of PRD and clinical features mimicking infections and malignancy (5, 6). Avoidable disability acquired in childhood due to PRD has lifelong medical, social, and economic burden on patients, families, and healthcare systems (1, 7).

Juvenile dermatomyositis (JDM), the most common idiopathic inflammatory myopathy affecting children, is a multisystem, autoimmune-mediated disease. JDM primarily causes proximal muscle weakness and pathognomonic cutaneous lesions (8, 9) but can also involve the joints, gastrointestinal tract, heart, lungs, and, more rarely, the kidneys, eyes, and central nervous system (10). JDM is often a chronic, highly morbid disease with high rates of permanent damage ranging from 50-79% (11, 12, 13). Due to its morbidity, understanding the burden of disease and long-term prognostic implications warrants particular attention in LMIC.

Several JDM studies have been reported in low middle-income countries (2, 3, 6, 10, 14, 15, 16, 17, 18, 19), though many are descriptive small single-center reports (2, 3, 6, 14, 15, 19). Several found higher mortality rates than those reported in North American and European studies (2, 17, 18). There have been few African studies; the majority are single-patient case reports (19, 20, 21, 22, 23) or a few JDM patients among larger cohorts of other PRD (4, 5, 24). To date, there are only three African case series from just 2 countries describing 46 South African (25, 26) and 134 Egyptian (27) children with JDM. These studies demonstrated high rates of severe disease manifestations both at diagnosis and throughout disease course, including cutaneous ulceration (27), global weakness, calcinosis, vasculitis, and pulmonary involvement (25, 26).

Calcinosis and interstitial lung disease (ILD) can be associated with severe disease in JDM, with calcinosis associated with increased morbidity (28) and ILD a risk factor for mortality (9). The South African case series found higher rates of calcinosis and ILD compared not only to those reported in studies of the Childhood Arthritis and Rheumatology Research Alliance (CARRA) JDM cohort (8) but also studies from other middle-income countries (3, 6, 14, 15, 17).

In high-income countries, particularly in North America and Europe, JDM outcomes have improved significantly due to early, aggressive treatment (29, 30), with mortality rates less than 1-3% (9, 31, 32), down substantially from 33% preceding corticosteroid use (9, 33). In contrast, mortality rates in studies from LMIC range from 11% in India (2) to 8-50% in Africa (4, 25, 26). These high mortality rates may be due to delays in diagnosis and treatment, late referrals to pediatric rheumatologists, financial constraints limiting medication adherence, and increased risk of infection (2).

While data is limited, existing reports seem to highlight severity of JDM in African patients. To our knowledge, there has not been a review of JDM across the African continent beyond reports from single centers. We suspect that existing literature does not fully capture the burden of disease. The objective of this survey is to better understand the provider experience of clinical burden of JDM in Africa and access to diagnostic tools and therapies by obtaining an overview of JDM and comparing this to data in previously published work.

**Methods**

*Survey design and administration*

An electronic, cross-sectional survey was designed by the authors using Qualtrics. It consisted of 29 questions in multiple choice, checkbox, and free-text format, with 5 additional questions to assess interest in future collaborative research efforts. Participants were excluded if they did not manage patients with JDM. To capture respondent demographic and geographic information, participants were queried about their role and practice setting. They could indicate the city and country of their clinical practice and the name of their institution; however, these questions were optional to protect anonymity. Participants were asked to indicate whether they were the only physician managing patients with JDM at their center or if there were multiple physicians to ensure that each child with JDM was included only once. Participants who were the sole physician managing children with JDM at their center answered the survey individually, accounting for all JDM patients at their center. At centers with two or more physicians managing children with JDM, participants were requested to either a) coordinate with other providers at their center and be the sole respondent for their center, with their responses reflective of all patients with JDM managed at their center; or b) indicate that they answered the survey individually with their responses reflective of only patients they manage but not necessarily reflective of all patients at their center.

We aimed to understand the burden of JDM in Africa, thus participants were queried about the total number of JDM patients followed at their center currently or within the last 10 years but no longer actively followed. They were also queried about reasons for discontinuation of care. They were then asked to indicate how many of these patients achieved clinically inactive disease while on medication and remission off medications. Clinically inactive disease was defined as “lack of evidence of myositis disease activity as assessed by global and extra-muscular assessments, stable muscle strength and function, and normal muscle enzyme levels”, as per the International Myositis Assessment & Clinical Studies (IMACS) criteria for lack of evidence of active myositis (34). Remission was defined as “clinically inactive disease while not receiving any drug therapy for a 6-month continuous period”, as per the IMACS 2005 definition (34). In order to understand the spectrum of disease manifestations and outcomes, participants were queried about the number of patients who developed calcinosis or ILD and if any died. Respondents were queried about resources used to diagnose and/or monitor JDM generally, resources used specifically to diagnose and/or monitor calcinosis and ILD, and medications used to treat JDM. For each, respondents were first asked which resources were available at their center, regardless of access and/or cost-related issues. They were then asked to indicate which of these available resources were typically used after considering access and/or cost-related issues. Finally, respondents were queried about challenges encountered in managing children with JDM.

The survey was available in English and French given that these serve as the official and/or commonly spoken languages in 49 of the 54 African countries; in the other 5 countries (São Tomé and Príncipe, Mozambique, Guinea-Bissau, Cape Verde, and Angola), Portuguese and/or native African languages serve as the official and/or commonly spoken languages (35). The survey was initially designed in English and translated into French using a certified medical translator. The survey was reviewed and revised by all authors prior to translation and distribution. A copy of the survey is available as Supplementary File 1.

The survey was distributed via WhatsApp groups to members of the African League of Associations for Rheumatology (AFLAR; n=233) and the Paediatric Society of the African League Against Rheumatism (PAFLAR; n=130) between November 2022 and January 2023. WhatsApp was selected given that it is used for communication between AFLAR and PAFLAR members and allows for calculation of response rate given known number of participants in each group (36); WhatsApp has been used for survey link distribution in several LMIC given that is a common method of communication (36, 37, 38). AFLAR and PAFLAR members include physicians managing rheumatological diseases at the clinical and/or research level, paramedical professionals (including physiotherapists, nurses, biomedical engineers, and technicians), residents, and medical students.

Participant agreement to participate in the survey was obtained prior to initiating the survey. Survey responses remained anonymous unless participants chose to share their contact information for the purpose of future collaboration. Participation was voluntary, and no compensation was provided. Albert Einstein College of Medicine Institutional Review Board (IRB) approval was obtained (approval number 2022-13864) with waiver of informed consent.

*Statistical analysis*

Statistical analysis was performed using STATA software, version 17.0. Individual responses were pooled together by country. All variables were examined to identify missing data and potential data entry errors. Descriptive statistics were applied to evaluate responses, which were summarized as frequencies for categorical variables; percentages were also reported when the total number of patients was reported by all participants of a country.

**Results**

*Respondent characteristics*

Forty-three individuals started the survey (12% of the 363 AFLAR and PAFLAR members who received the survey), of whom 37 (37/43; 86%) were eligible to complete the survey (participant provided consent and manages children with JDM). Among these 37 respondents eligible to complete the survey, 11 (11/37; 30%) did not proceed further with the survey and 6 (16%) only indicated their country of practice, therefore these 17 (46%) respondents were excluded, leaving 20 (54%) respondents for the remainder of the analyses. Of these 20 respondents, 4 (20%) partially and 16 (80%) fully completed the survey. The majority of respondents included in analysis (19/20; 95%) were adult and/or pediatric rheumatologists, with 1 general pediatrician also completing the survey (5%) (Figure 1). Most respondents practice in an academic hospital setting (n=17; 85%), with a smaller number practicing in community hospitals (n=4; 20%) and/or private clinics (n=8; 40%); 6 respondents (30%) practice in multiple settings and therefore total percentages of responses to this question exceed 100. Respondents represented all 5 African regions, with most from Northern Africa (n=12; 60%) and smaller representation in Eastern Africa (n=3; 15%), Central Africa (n=3; 15%), Western Africa (n=1; 5%), and Southern Africa (n=1; 5%) (Figure 2, Table 1). Six respondents (30%) were the sole providers at their center; of the remaining respondents who were not sole providers, 12 respondents (60%) completed the survey individually, answering only about their unique patients, and 2 respondents (10%) coordinated with other providers and completed the survey based on all patients at their center.

*Patient characteristics*

Respondents described at least 216 children with JDM within the last 10 years, of which 151 are followed currently and 65 are no longer actively followed. Several respondents from Egypt, Libya, and Tunisia were unable to provide the total number of patients followed: 3 respondents were unable to provide an estimate of the total number of children seen currently and 5 respondents were unable to provide an estimate of the total number of children seen previously within the last 10 years. In these cases, percentages were not able to be calculated. Respondents reported several reasons for discontinuation of care, including loss to follow-up (50% of respondents), death of patient (22%), patient older than the age cutoff for the center and/or was referred to an adult provider (22%), family could no longer afford medical care (17%), family moved (11%), and other reasons (22%) including remission off treatment and transfer to private clinic.

Table 1 depicts the prevalence of various clinical outcomes among respondents by country. Clinically inactive disease was achieved by 25-100% of children and remission was achieved by 21-46% of children. The prevalence of severe manifestations (defined as calcinosis, ILD, death) varied among countries. Prevalence of calcinosis ranged widely, from 0% in Nigeria to 100% in the Democratic Republic of the Congo. ILD was reported to be present in 0-32% of children. Respondents from most countries reported no patient deaths; however, 6 children (14%) in Kenya and 2 children (29%) in Zambia died. Respondents from Egypt and Libya also reported deaths, but percentages were not able to be calculated due to unknown number of total patients, and response was missing from the respondent in the Democratic Republic of the Congo (Table 1).

Table 2 shows the availability of and access to diagnostic tools and medications. Among resources used to diagnose JDM generally, irrespective of access and/or cost, all were available to at least 50% of respondents except for von Willebrand factor (vWF) antigen, which was available to only 4 respondents (22%). However, once access and/or cost were considered, all available resources were reported as typically used by fewer participants with the exception of muscle enzymes (creatinine kinase [CK], aldolase, lactate dehydrogenase [LDH], and/or aspartate aminotransferase [AST]), which were both available and accessible to all respondents. This discrepancy between availability and accessibility of diagnostic tools was also noted for all resources used specifically to diagnose and/or monitor calcinosis and ILD. Oral corticosteroids and methotrexate were the only medications that were both equally available and accessible among all respondents. For all other medications, fewer respondents indicated that they were typically used after considering access and/or cost. Among the 27 diagnostic tools and medications included in the survey, 13 (48%) were accessible to ≤50% of respondents; the majority (9/13; 69%) of these diagnostic tools or medications were only accessible in Northern (Egypt, Tunisia, Libya, Morocco) or Southern (South Africa) African countries. These are highlighted in Table 2 and include vWF antigen, myositis-specific antibodies (MSA) or myositis-associated antibodies (MAA), muscle biopsy, electromyography (EMG), magnetic resonance imaging (MRI), and ultrasound among the diagnostic resources; and hydroxychloroquine/chloroquine, cyclosporine, cyclophosphamide, rituximab, tumor necrosis factor (TNF) inhibitors, abatacept, and janus kinase (Jak) inhibitors among medications. Despite the fact that there is no cost associated with physical exam or disease assessment tools, these were not indicated as being available or accessible to all respondents.

Respondents identified several challenges in managing children with JDM. Specifically, respondents identified delayed presentation to care (12 of 16 respondents; 75%), unfamiliarity of JDM among caregivers (11/16; 69%), lack of familiarity with JDM among other medical providers (7/16; 44%), caregivers and/or patients refused to initiate or continue treatment due to stigma surrounding JDM (4/16; 25%), and limited availability of resources – including medications (9/16; 56%), non-medical therapies such as physical and occupational therapy (5/16; 31%), and diagnostic tools (4/16; 25%). Additionally, general physicians caring for pediatric rheumatology patients expressed their own limited familiarity with JDM.

**Discussion**

Our survey of AFLAR and PAFLAR members identified at least 216 children with JDM cared for across the African continent within the last 10 years. Respondents were mostly rheumatologists practicing in academic hospitals and represented all 5 regions of Africa. While prevalence of severe disease outcomes varied among countries, prevalence was as high as 100% for calcinosis, 32% for ILD, and 29% for mortality. Respondents identified limitations in accessibility of medications, and almost half of all queried resources were accessible to ≤50% of respondents, mostly in Northern and Southern Africa.

To date, only 196 children with JDM reviewed within the last 25 years have been reported in the literature, with the vast majority described in case series from just 2 countries, Egypt (134 children) and South Africa (46 children) (3, 25, 26); the remainder have been described in single-patient case reports (20, 21, 22, 23) or a few JDM patients among larger cohorts of other pediatric rheumatic diseases or idiopathic inflammatory myopathies (4, 5, 24). The 216 children with JDM seen within only the last 10 years described in our survey exceeds what has been reported in the past 25 years. Given that as of 2021, there were known rheumatology centers in only 10 of the 54 African countries (39), this number still likely vastly underestimates true prevalence as our survey did not capture all known patients with JDM and does not account for the likely many children who remain undiagnosed. While epidemiologic studies of JDM have focused mostly on incidence, with less known about prevalence, one study in France determined the prevalence of JDM to be 3.78 per 100,000 children age 0-16 years, with similar incidence to what is reported in the literature (40). There are an estimated approximately 650 million children age 0-17 years in Africa as of 2021 (41); assuming similar prevalence, there may be 24,000-25,000 children with JDM on the continent. While our understanding of the prevalence of JDM in Africa is limited, the findings of our survey highlight that existing literature likely greatly underestimates the true burden of JDM on the continent.

Overall, the prevalence of severe disease manifestations and outcomes in several countries is consistent with what is reported in the literature. However, in other countries represented in our survey, observed prevalence was higher than what has been reported. Prevalence of calcinosis ranges widely and is thought to depend on various factors, including MSA and/or MAA (29), diagnostic delays, disease duration, and Black race (28, 42); studies of national and/or international registries estimate prevalence between 12-47% (8, 11, 12, 16, 17, 43), though a single-center case series in South Africa found a prevalence of 71% (26). Respondents to our survey generally identified similar prevalence of calcinosis, though the prevalence reported in South Africa (50%) and Democratic Republic of the Congo (100%) was higher than estimates from national and/or international registries (8, 11, 12, 16, 17, 43). Prevalence estimates of ILD in JDM range from 7-19% (44); while prevalence estimates in Kenya, Zambia, and Cameroon were consistent, ILD was reported in 25% of Nigerian and 32% of Moroccan children. Finally, mortality estimates are 1-3%, though these are mostly based on studies from high-income countries (9, 31, 32). While most respondents reported similar estimates, higher mortality was reported in Kenya (14%) and Zambia (29%).

When exploring availability and accessibility of diagnostic tools and medications commonly used in the management of JDM, regional differences were noted. Forty-eight percent of the queried resources were accessible to 50% or fewer of respondents, mostly in Northern or South Africa. This reflects regional healthcare disparities on the continent, with Northern and Southern African countries having the highest Healthcare Access and Quality (HAQ) Index in the continent (45). Interestingly, diagnostic tools without any associated cost, namely disease assessment tools (including the Childhood Myositis Assessment Scale [CMAS], Manual Muscle Testing (MMT), and Physician and Patient/Parent Global Activity Visual Analogue Scale [VAS], among others) and physical exam to assess calcinosis, were not indicated as being universally available or accessible, despite their important role in the management of JDM (46). This may reflect limitations in time required to utilize these tools and/or lack of familiarity with their use; increasing their use may reflect a cost-effective way to improve the diagnosis and management of JDM.

Our study had several notable strengths. To our knowledge, this is the first study to explore the scope of JDM across the African continent as a whole, including an overview of availability and accessibility of diagnostic tools and medications. Furthermore, we had representation from all 5 regions of the continent, ensuring our findings were broadly representative of the continent. Our respondents identified more children with JDM seen in the last 10 years than have been represented in all the literature to date in the last 25 years, suggesting that the prevalence of JDM is not fully captured by existing publications. Furthermore, as of 2021, there were known rheumatology centers in only 10 of the 54 African countries (39); however, rheumatologists from 5 countries not included in this report (Cameroon, Democratic Republic of the Congo, Morocco, Tunisia, and Zambia) responded to our survey. This suggests that there may have been interim growth in the rheumatology workforce and/or these practitioners were not captured by the 2021 report. Given that our survey was distributed to all members of AFLAR and PAFLAR via WhatsApp groups, we were able to minimize sampling bias. Finally, our survey was distributed in both English and French, the official and/or commonly spoken language in 90% of African countries, thereby ensuring equity in participation.

Despite these strengths, our study also had several limitations inherently seen with surveys. While the potential reach of our survey was broad, only a small proportion of recipients initiated the survey; while over half of those who started the survey partially or fully completed it, overall the number of respondents was small. Furthermore, not all countries were represented by respondents, therefore our understanding of the scope of JDM across the entire African continent, while an improvement on existing literature, remains limited. As a result, while our survey provides improved insight into the burden of JDM in Africa, it still likely vastly underestimates the true prevalence of disease since we 1) did not capture all known patients with JDM and 2) did not account for the, likely many, children with JDM who remain undiagnosed. While we strived to count unique JDM patients by asking respondents who share patients at a single center to coordinate their responses, it is possible that the same patient may have been included in multiple survey responses. Though most African countries officially and/or commonly use English and/or French, the remaining 5 countries speak Portuguese and/or native African languages, therefore providers from these countries may not have been able to participate in the survey. Given that this was a survey, we did not analyze outcomes based on MSA or MAA, the distribution of which may have influenced the prevalence of outcomes such as calcinosis and ILD. Finally, given that we inquired about children seen within the last 10 years but who are not currently followed, there may have been recall bias; respondents may have been more likely to remember severe outcomes (leading to overestimation), and their estimation of the number of patients with JDM may not have been fully accurate since we did not ask them to confirm with medical record review as this would have been cumbersome and reduced response rate.

Importantly, this survey identifies not only existing limitations in care of children with JDM but also highlights opportunities to improve their care and outcomes. Our survey demonstrates that existing literature underestimates disease burden; furthermore, the number of children identified in our survey is still likely a vast underestimation of disease prevalence. Understanding epidemiology of disease is critical and can be achieved through the establishment of international patient registries (46), as has recently been successfully achieved by PAFLAR for juvenile idiopathic arthritis (JIA) (47). Limited familiarity of JDM among healthcare providers leads to missed diagnoses and inadequate management, challenges that have been identified not only in our survey but in prior work (1, 48). While increasing the pediatric rheumatology workforce is a crucial long-term goal, educating general practitioners, pediatricians, allied healthcare professionals, and community health workers is perhaps a more achievable goal that can have widespread impact on recognition and initial management of disease. A variety of strategies have been proposed, including local education campaigns, use of free and accessible online educational materials such as Pediatric Musculoskeletal Matters, and use of telemedicine to improve access to care (1, 48). Our survey also demonstrates that caregivers are unfamiliar with JDM and that there is stigma surrounding disease; educating caregivers and patients and providing access to support networks and resources are critical steps in improving the patient and caregiver experience. Finally, our survey identifies limited availability of and access to a variety of diagnostic tools and medications. There are efforts underway to revise and expand the World Health Organisation (WHO) Essential Medicines List for pediatric rheumatology. This list informs policy makers about which medications are most important for the management of PRD, particularly in LMIC; however, the list currently lacks many critical medicines needed to appropriately manage PRD (49, 50). These efforts require funding and advocacy but can have huge impacts on improving the equity of care for children globally.

**Conclusions**

In conclusion, our survey of African providers identified a greater number of children with JDM than what is reflected in existing literature, supporting the notion that limited existing reports do not reflect disease rarity on the continent but rather underreporting and undercounting true disease burden (1, 4). The potential differences in JDM manifestations associated with increased morbidity and mortality warrant further systematic study; if there are indeed differences in African patients, it is critical to understand why these differences occur and to develop tailored management guidelines that account for these differences. The inclusion of children and providers from African countries and other LMIC in global collaborative research is critical to ensure equity and generalizability of research and improve outcomes.

**List of abbreviations**

Pediatric rheumatic diseases (PRD); low and middle-income countries (LMIC); juvenile dermatomyositis (JDM); interstitial lung disease (ILD); Childhood Arthritis and Rheumatology Research Alliance (CARRA); International Myositis Assessment & Clinical Studies (IMACS); African League of Associations for Rheumatology (AFLAR); Paediatric Society of the African League Against Rheumatism (PAFLAR); Institutional Review Board (IRB); von Willebrand factor (vWF); creatinine kinase (CK); lactate dehydrogenase (LDH); aspartate aminotransferase (AST); myositis-specific antibodies (MSA); myositis-associated antibodies (MAA); electromyography (EMG); magnetic resonance imaging (MRI); tumor necrosis factor (TNF); januse kinase (Jak); juvenile idiopathic arthritis (JIA); World Health Organisation (WHO)

**Declarations**

**Ethics approval and consent to participate**

Albert Einstein College of Medicine Institutional Review Board (IRB) approval was obtained (approval number 2022-13864) with waiver of informed consent.

**Consent for publication**

Not applicable.

**Availability of data and materials**

The datasets used and/or analyzed during the current study are available from the corresponding author on reasonable request.

**Competing interests**

The authors declare that they have non-competing interests.

**Funding**

The Children’s Hospital at Montefiore Fellow Grant. Dr. Laura B. Lewandowski was funded by the National Institute of Arthritis Musculoskeletal and Skin Diseases Intramural research program.

**Authors’ contributions**

JP, LBL, DMW, KW, CS, and AM conceptualized the study; JP drafted the survey; CS, KW, and AM distributed the survey; JP performed data analysis; JP drafted the manuscript; all authors read and approved the final manuscript.

**Acknowledgements**

This research was supported by the NIH/National Center for Advancing Translational Science (NCATS) Einstein-Montefiore CTSA Grant Number TLITR001072.

**References**

1. Scott C, Sawhney S, Lewandowski LB. Pediatric Rheumatic Disease in Lower to Middle-Income Countries: Impact of Global Disparities, Ancestral Diversity, and the Path Forward. Rheum Dis Clin North Am. 2022;48(1):199-215.

2. Singh S, Suri D, Aulakh R, Gupta A, Rawat A, Kumar RM. Mortality in children with juvenile dermatomyositis: two decades of experience from a single tertiary care centre in North India. Clin Rheumatol. 2014;33(11):1675-9.

3. Al-Mayouf SM, AlMutiari N, Muzaffer M, Shehata R, Al-Wahadneh A, Abdwani R, et al. Phenotypic characteristics and outcome of juvenile dermatomyositis in Arab children. Rheumatol Int. 2017;37(9):1513-7.

4. Furia FF, Godfrey E, Mwamanenge N, Swai P. Spectrum of paediatric rheumatic disorders at a tertiary hospital in Tanzania. Pediatr Rheumatol Online J. 2020;18(1):30.

5. Olaosebikan BH, Adelowo OO, Animashaun BA, Akintayo RO. Spectrum of paediatric rheumatic diseases in Nigeria. Pediatr Rheumatol Online J. 2017;15(1):7.

6. Prasad S, Misra R, Agarwal V, Lawrence A, Aggarwal A. Juvenile dermatomyositis at a tertiary care hospital: is there any change in the last decade? Int J Rheum Dis. 2013;16(5):556-60.

7. Martin N, Krol P, Smith S, Murray K, Pilkington CA, Davidson JE, et al. A national registry for juvenile dermatomyositis and other paediatric idiopathic inflammatory myopathies: 10 years' experience; the Juvenile Dermatomyositis National (UK and Ireland) Cohort Biomarker Study and Repository for Idiopathic Inflammatory Myopathies. Rheumatology (Oxford). 2011;50(1):137-45.

8. Robinson AB, Hoeltzel MF, Wahezi DM, Becker ML, Kessler EA, Schmeling H, et al. Clinical characteristics of children with juvenile dermatomyositis: the Childhood Arthritis and Rheumatology Research Alliance Registry. Arthritis Care Res (Hoboken). 2014;66(3):404-10.

9. Huber A, Feldman BM. Long-term outcomes in juvenile dermatomyositis: how did we get here and where are we going? Curr Rheumatol Rep. 2005;7(6):441-6.

10. Guseinova D, Consolaro A, Trail L, Ferrari C, Pistorio A, Ruperto N, et al. Comparison of clinical features and drug therapies among European and Latin American patients with juvenile dermatomyositis. Clin Exp Rheumatol. 2011;29(1):117-24.

11. Sanner H, Gran JT, Sjaastad I, Flatø B. Cumulative organ damage and prognostic factors in juvenile dermatomyositis: a cross-sectional study median 16.8 years after symptom onset. Rheumatology (Oxford). 2009;48(12):1541-7.

12. Mathiesen PR, Zak M, Herlin T, Nielsen SM. Clinical features and outcome in a Danish cohort of juvenile dermatomyositis patients. Clin Exp Rheumatol. 2010;28(5):782-9.

13. Rider LG, Lachenbruch PA, Monroe JB, Ravelli A, Cabalar I, Feldman BM, et al. Damage extent and predictors in adult and juvenile dermatomyositis and polymyositis as determined with the myositis damage index. Arthritis Rheum. 2009;60(11):3425-35.

14. Chickermane PR, Mankad D, Khubchandani RP. Disease patterns of juvenile dermatomyositis from Western India. Indian Pediatr. 2013;50(10):961-3.

15. Chowdhary V, Wakhlu A, Agarwal A, Misra R. Outcome in juvenile dermatomyositis. Indian Pediatr. 2002;39(10):931-5.

16. Ravelli A, Trail L, Ferrari C, Ruperto N, Pistorio A, Pilkington C, et al. Long-term outcome and prognostic factors of juvenile dermatomyositis: a multinational, multicenter study of 490 patients. Arthritis Care Res (Hoboken). 2010;62(1):63-72.

17. Sato JO, Sallum AM, Ferriani VP, Marini R, Sacchetti SB, Okuda EM, et al. A Brazilian registry of juvenile dermatomyositis: onset features and classification of 189 cases. Clin Exp Rheumatol. 2009;27(6):1031-8.

18. Sallum AM, Pivato FC, Doria-Filho U, Aikawa NE, Liphaus BL, Marie SK, et al. Risk factors associated with calcinosis of juvenile dermatomyositis. J Pediatr (Rio J). 2008;84(1):68-74.

19. Mussa F, Nalitolela N, Fredrick F. An 8-year-old-girl with juvenile dermatomyositis and autoimmune thyroiditis in Tanzania: a case report. J Med Case Rep. 2021;15(1):632.

20. Grijsen ML, McHaile D, Geut I, Olomi R, Nwako M, Requena L, et al. Juvenile dermatomyositis in a 4-year-old Kenyan girl. Clin Case Rep. 2017;5(2):134-8.

21. Adelowo O, Nwankwo M, Olaosebikan H. Juvenile dermatomyositis in a Nigerian girl. BMJ Case Rep. 2014;2014.

22. Aliu R, Aliyu LI, Obiagwu PN, Olatoke L, Ebisike JK. Juvenile dermatomyositis in a 14-year old Nigerian girl. Arch Clin Cases. 2020;7(1):5-9.

23. Mandengue CE, Nouedoui C, Atangana PJ. [Unrecognized juvenile dermatomyositis complicated by calcinosis universalis: a case report from Cameroon]. Med Sante Trop. 2013;23(4):458-61.

24. Chinniah KJ, Mody GM. The spectrum of idiopathic inflammatory myopathies in South Africa. Clin Rheumatol. 2021;40(4):1437-46.

25. Okong'o LO, Esser M, Wilmshurst J, Scott C. Characteristics and outcome of children with juvenile dermatomyositis in Cape Town: a cross-sectional study. Pediatr Rheumatol Online J. 2016;14(1):60.

26. Faller G, Mistry BJ, Tikly M. Juvenile dermatomyositis in South African children is characterised by frequent dystropic calcification: a cross sectional study. Pediatr Rheumatol Online J. 2014;12:2.

27. El-Garf K, El-Garf A, Salah S, Marzouk H, Farag Y, Mostafa N. A juvenile dermatomyositis: demographics, characteristics and disease outcome in an Egyptian cohort. Clin Exp Rheumatol. 2022;40(2):450-6.

28. Phillippi K, Hoeltzel M, Byun Robinson A, Kim S. Race, Income, and Disease Outcomes in Juvenile Dermatomyositis. J Pediatr. 2017;184:38-44.e1.

29. Wu Q, Wedderburn LR, McCann LJ. Juvenile dermatomyositis: Latest advances. Best Pract Res Clin Rheumatol. 2017;31(4):535-57.

30. Kim H, Huber AM, Kim S. Updates on Juvenile Dermatomyositis from the Last Decade: Classification to Outcomes. Rheum Dis Clin North Am. 2021;47(4):669-90.

31. Smith S, Juggins A, Evans S, Pilkington C. What is the mortality of Juvenile Dermatomyositis (JDM) in the modern treatment era. Pediatric rheumatology online journal. 2008;6(Suppl 1):P218-P.

32. Huber A, Feldman BM. An update on inflammatory myositis in children. Curr Opin Rheumatol. 2013;25(5):630-5.

33. Bitnum S, Daeschner CW, Jr., Travis LB, Dodge WF, Hopps HC. DERMATOMYOSITIS. J Pediatr. 1964;64:101-31.

34. Oddis CV, Rider LG, Reed AM, Ruperto N, Brunner HI, Koneru B, et al. International consensus guidelines for trials of therapies in the idiopathic inflammatory myopathies. Arthritis Rheum. 2005;52(9):2607-15.

35. Official and Spoken Languages of African Countries [Available from: <https://www.nationsonline.org/oneworld/african_languages.htm>.

36. Oluwadiya KS, Olasinde AA, Adeoti AO, Adeoye O, Oluwadiya IO, Kadiri IA. The high cost of healing and teaching: a cross-sectional survey of burnout among academic physicians in Nigeria. BMC Health Serv Res. 2023;23(1):1357.

37. Manji K, Hanefeld J, Vearey J, Walls H, de Gruchy T. Using WhatsApp messenger for health systems research: a scoping review of available literature. Health Policy Plan. 2021;36(5):594-605.

38. Sianturi EI, Latifah E, Pane M, Perwitasari DA, Satibi, Kristina SA, et al. Knowledge, empathy, and willingness to counsel patients with HIV among Indonesian pharmacists: a national survey of stigma. AIDS Care. 2022;34(1):21-8.

39. Migowa AN, Hadef D, Hamdi W, Mwizerwa O, Ngandeu M, Taha Y, et al. Pediatric rheumatology in Africa: thriving amidst challenges. Pediatr Rheumatol Online J. 2021;19(1):69.

40. Moegle C, Severac F, Lipsker D. Epidemiology of juvenile dermatomyositis in Alsace. Br J Dermatol. 2020;182(5):1307-8.

41. United Nations DoEaSA, Population Division. World Population Prospects 2022 2023 [updated 2022. Available from: <https://population.un.org/wpp/>.

42. Hoeltzel MF, Becker ML, Robinson AB, Feldman BM, Huber A, Reed AM, et al. Race is a risk factor for calcinosis in patients with JDM – early results from the CARRAnet registry study. Pediatric Rheumatology. 2012;10(1):A65.

43. McCann LJ, Juggins AD, Maillard SM, Wedderburn LR, Davidson JE, Murray KJ, et al. The Juvenile Dermatomyositis National Registry and Repository (UK and Ireland)--clinical characteristics of children recruited within the first 5 yr. Rheumatology (Oxford). 2006;45(10):1255-60.

44. Sanner H, Aaløkken TM, Gran JT, Sjaastad I, Johansen B, Flatø B. Pulmonary outcome in juvenile dermatomyositis: a case-control study. Ann Rheum Dis. 2011;70(1):86-91.

45. Healthcare Access and Quality Index based on mortality from causes amenable to personal health care in 195 countries and territories, 1990-2015: a novel analysis from the Global Burden of Disease Study 2015. Lancet. 2017;390(10091):231-66.

46. Bellutti Enders F, Bader-Meunier B, Baildam E, Constantin T, Dolezalova P, Feldman BM, et al. Consensus-based recommendations for the management of juvenile dermatomyositis. Ann Rheum Dis. 2017;76(2):329-40.

47. Migowa AN HW, Hashad S et al. . The Clinical-Epidemiological Profile of Juvenile Idiopathic Arthritis in Africa: Data from the Paediatric Society of the African League Against Rheumatism (PAFLAR) Registry. PREPRINT (Version 1) available at Research Square [<https://doiorg/1021203/rs3rs-3918846/v1>]. 2024.

48. Migowa A, Bernatsky S, Ngugi A, Foster HE, Muriuki P, Lusambili A, et al. An iceberg I can’t handle: a qualitative inquiry on perceptions towards paediatric rheumatology among healthcare workers in Kenya. Pediatric Rheumatology. 2023;21(1):6.

49. Slamang W, Smith N, Scott C, Foster H. Revising the WHO Essential Medicines List for paediatric rheumatology update. Pediatr Rheumatol Online J. 2022;20(1):89.

50. Foster HE, Scott C. Update the WHO EML to improve global paediatric rheumatology. Nature Reviews Rheumatology. 2020;16(3):123-.

**Figure 1**

***Title: Respondent specialty***

***Legend:***

**
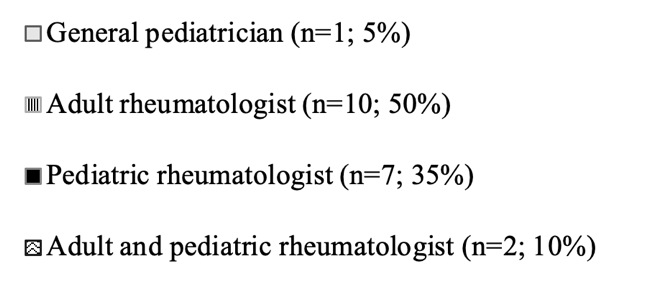
**

**Figure 2**

***Title: Respondent country of origin***

***Legend:***

***
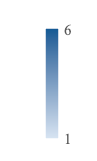
***
