# Supplementary material for: Exploring the clinical profiles and management of juvenile dermatomyositis in Africa: a survey of African rheumatology care providers
Source: Pediatr Rheumatol Online J. 2025 Jan 27;23:10. doi: 10.1186/s12969-024-01009-8 (PMC11771122; doi:10.1186/s12969-024-01009-8)
Supplement: Supplementary file 4 — Supplementary Material 4 [file 12969_2024_1009_MOESM4_ESM.docx]

January 18, 2024

To the Editors of *Pediatric Rheumatology,*

We present a survey of members of the African League of Associations for Rheumatology (AFLAR) and Paediatric Society of the African League Against Rheumatism (PAFLAR) to understand the provider experience of clinical burden of juvenile dermatomyositis (JDM) in Africa and access to diagnostic tools and therapies. There are limited studies of JDM in low and middle-income countries (LMIC), including Africa; they demonstrate high prevalence of severe outcomes, but African children with JDM have not been systematically studied nor have the experiences of providers caring for these children been explored.

Our study is the first to obtain a broad overview of JDM across the African continent and to explore provider experiences in caring for children with JDM. Our survey identified more children with JDM across the continent in the last 10 years than all the children with JDM reported in the literature in the past 25 years and still likely vastly underestimates true prevalence. While the prevalence of some severe disease outcomes is consistent with prevalence generally reported in the literature, several respondents reported prevalence higher than what has been observed in other studies to date. Respondents also identified limitations in access to diagnostic tools and medications, with regional disparities observed. Our study highlights the need to systemically study JDM in children from Africa and other LMIC to explore possible differences in disease severity. Our study also demonstrates the need to include children and providers from African countries and other LMIC in global collaborative research to ensure increased equity and generalizability.

All authors affirm that:

1. They have no competing interests.
2. There are no issues relating to journal policies.
3. They have approved the manuscript for submission.
4. The required ethical approvals have been obtained.
5. They have given necessary attention to ensure integrity of the work.
6. They agree to bear the applicable publication charges.
7. The content of the manuscript has not been published. It is not submitted, nor under consideration, for publication elsewhere.

Thank you for your kind consideration of this important and instructive study for publication.

Sincerely,


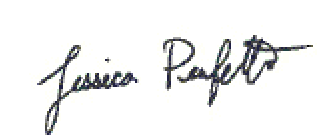


Jessica Perfetto, M.D.

Corresponding Author

Department of Pediatrics, Division of Rheumatology

The Children’s Hospital at Montefiore

Address: 3334 Bainbridge Avenue, Bronx, New York 10467

Office telephone: (718) 696-2892

Email: jperfetto@montefiore.org
