# Supplementary material for: Exploring the clinical profiles and management of juvenile dermatomyositis in Africa: a survey of African rheumatology care providers
Source: Pediatr Rheumatol Online J. 2025 Jan 27;23:10. doi: 10.1186/s12969-024-01009-8 (PMC11771122; doi:10.1186/s12969-024-01009-8)
Supplement: Supplementary file 2 — Supplementary Material 2 [file 12969_2024_1009_MOESM2_ESM.docx]

Reviewer reports:

Reviewer #1: Dear authors, we recommend that you make the following adjustments to the manuscript.
1. Consider renaming the title of the manuscript, since the current title does not reflect its contents

*We have removed “outcomes” and replace it with “management” to better capture the focus on both clinical manifestations but also resources and challenges in care.*

2. In the methods section of the abstract, indicate the total number of respondents who were asked to participate in the survey and those who took part.

*We had already included the number of AFLAR and PAFLAR members the survey was sent to in the methods of the abstract but we also added the total number of members (363) to page 2, line 37. The revised section is page 2, lines 37-39: “A survey (available in English and French) was distributed via WhatsApp to 363 total members of the African League of Associations for Rheumatology (AFLAR; n=233) and Paediatric Society of the African League Against Rheumatism (PAFLAR; n=130)”. The results section contains the number of participants who participated in the survey (37 participants who provided consent and indicate that they manage JDM, therefore were eligible to complete the survey). Please let us know if this information should be clarified.*

*We also clarified the denominators in the results of the main paper: page 9, line 203; page 9, line 207.*

3. In the abstract in the results section, the number of respondents is indicated as a fraction; please check the correctness of the data.

*We do not see a fraction for number of participants; we verified the percentages are correct. Of the 363 providers who received the survey, 43 (12%) started it. Of these 43, 37 (86%) provided consent/manage patients and were therefore eligible to complete the survey. We tried to be clear what was being used as the denominator, and explain in more detail in the body of the paper, but please let us know if this remains unclear. We tried to clarify this on page 2, lines 45-47: “Forty-three (12%) of the 363 providers who received the survey started it. Among the 43 who started the survey, 37 (86%) provided consent and manage JDM patients; of these 37 providers, 4 (11%) partially and 16 (43%) fully completed the survey.”*

4. We ask you to avoid abbreviations that have not been introduced previously (for example, ILD).

*Thank you for noting this; it has been corrected and ILD is now defined as interstitial lunge disease (page 2, line 50).*

5. Describe in more detail the data on the prevalence of severe manifestations of the disease in the abstract; when describing data on mortality, indicate from what total number the percentage is calculated.

*Thank you for this suggestion. We added maximum prevalence for calcinosis and ILD (page 2, lines 49-50). We also added the denominators for mortality in Kenya and Zambia (Page 2, lines 50-51).*

6. Consider updating reference #34. The last consensus guidelines for the treatment of JDM was published in 2017 by Enders.

*This reference was used only on pages 6-7 (lines 152-158) – in this paper from 2005, consensus definitions of clinically inactive disease and remission (that we used in our paper) were defined. Thank you for sharing the Enders 2017 paper – we have added it as a reference to page 14, line 326 to support the importance of these diagnostic tools and to page 16, line 373 to highlight the importance of patient registries.*

7. In the main part of the manuscript, it is more correct to present data on the reasons for discontinuation of treatment in the form of the number (and/or %) of patients. When describing such a parameter as a reason for stopping treatment, indicating the number of respondents, in our opinion, is not informative.

*This is an excellent point. Unfortunately, in the survey we did not ask respondents to indicate the number of patients who discontinued care for each of those reasons. Instead, we asked providers generally what reasons their patients have had for discontinuing care. We hoped that this would provide a broad overview of common challenges faced to continuity of care.*

8. In the section on the problems of managing patients with JDM (lines 251-258), indicate the absolute numbers of indicators, which are presented as %. Describe who exactly is meant in this section: patients or respondents.

*We modified the text and also added the numbers and denominators to help clarify that these represent respondents rather than patients. This has been modified on page 11, lines 266-272.*

Reviewer #2: The paper is so interesting to all rheumatology community around the world

I would like to suggest if were possible, to add some additional information related to:
1. Type of affectation: one shot, relapses and remissions
2. Potential therapy adherence
3. Disability
4. Remission with or without treatment maintained
5. Some differences or not with other

*Thank you for your feedback. Unfortunately, we do not have more detailed data for these specific points as this was a general survey aimed to capture broad patient information. However, these are very interesting and important details to address in future work. While we did include information on remission off medication, unfortunately we do not know if it was sustained, and for how long.*

Editors comments
While this survey identifies a major gap in the diagnosis and management of this relatively common autoimmune condition in most of the African continent save two countries, it would be appropriate for the discussion to focus on possible solutions briefly

*Thank you for this suggestion – we have added a discussion on possible solutions at the end of the discussion (page 16-17, lines 368-392).*
